# Landscape of potential germline pathogenic variants in select cancer susceptibility genes in patients with adult‐type ovarian granulosa cell tumors

**DOI:** 10.1002/cam4.7340

**Published:** 2024-06-19

**Authors:** Rebekah M. Summey, Erica Gornstein, Brennan Decker, Kali C. Dougherty, Janet S. Rader, Elizabeth Hopp

**Affiliations:** ^1^ Division of Gynecologic Oncology, Department of Obstetrics and Gynecology Medical College of Wisconsin Milwaukee Wisconsin USA; ^2^ Foundation Medicine Institute Cambridge Massachusetts USA

**Keywords:** cancer genetics, cancer prevention, cancer risk factors, clinical cancer research

## Abstract

**Objective:**

The objective of this study was to assess the frequency of potential germline pathogenic variants that may contribute to risk of development of adult granulosa cell tumors (AGCT) given the paucity of germline testing guidelines for these patients.

**Methods:**

This was a retrospective cross‐sectional study analyzing comprehensive genomic profiling (CGP) results of AGCT with the *FOXL2* p.C134W mutation submitted to Foundation Medicine between 2012 and 2022. Cases with a potential germline pathogenic variant were identified by filtering single nucleotide variants and short indels by variant allele frequency (VAF) and presence in ClinVar for select cancer susceptibility genes. Odds ratios for AGCT risk were calculated compared to a healthy population.

**Results:**

Prior to analysis, 595 patients were screened and 516 with a somatic *FOXL2* p.C134W mutation were included. Potential germline pathogenic variants in a DNA repair‐related gene (*ATM*, *BRCA1*, *BRCA2*, *CHEK2*, *PALB2*, *PMS2*, *RAD51C*, or *RAD51D*) were found in 6.6% of *FOXL2*‐mutated AGCT. Potential germline pathogenic *CHEK2* variants were found in 3.5% (18/516) of AGCT patients, a rate that was 2.8‐fold higher than Genome Aggregation Database non‐cancer subjects (95% CI 1.8–4.6, *p* < 0.001). The founder variants p.I157T (38.9%, 7/18) and p.T367fs*15 (c.1100delC; 27.8%, 5/18) were most commonly observed. *CHEK2* VAF indicated frequent loss of the wildtype copy of the gene.

**Conclusions:**

These results support ongoing utilization of genomic tumor profiling and confirmatory germline testing for potential germline pathogenic variants. Further prospective investigation into the biology of germline variants in this population is warranted.

## Graphical abstract

Predicted germline mutations were found in 6.6% of sequenced, FOXL2‐mutated AGCT. Predicted germline CHEK2 were found in 3.5% of this cohort, 2.8 times higher than anticipated. Further prospective investigation into the biology of germline mutations in this population is warranted.

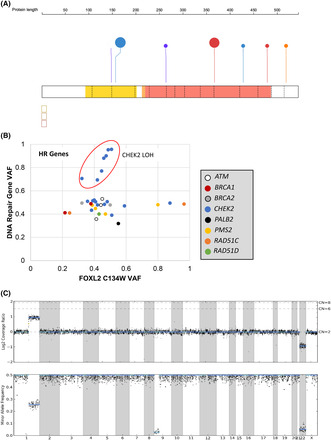

## 1 INTRODUCTION

Adult granulosa cell tumors (AGCT) are ovarian sex cord‐stromal tumors that occur at the site of female steroidogenesis. The need for improved biological understanding and treatment approaches is underscored by the fact that 50%–80% of patients die from recurrent disease.

The somatic mutational landscape of this tumor has been well described, with a nearly pathognomonic *FOXL2* p.C134W mutation in 85%–95% of cases, as well as less frequent recurrent *TERT* promoter (~40%), *KMT2D* (~17%), *CDH1* (~9%), and *TP53* (~5%) mutations.^1^, ^2^, ^3^ A recent study by Hillman et al. reported that *FOXL2* p.C134W was found in almost all of 423 patients' AGCTs sequenced by Foundation Medicine, but few currently actionable alterations were identified, emphasizing the critical need for precision treatment options for women with AGCT.^3^

Much less is known about germline predisposition to AGCT in comparison to other tumors of the ovary. Germline testing for heritable variants is now recommended for all patients with epithelial ovarian cancers, and both management strategies and treatments have been developed based on testing results.^4^ For nearly a decade, germline *BRCA1/2* pathogenic variants have been established indicators of platinum sensitivity and PARP inhibitor eligibility for these patients.^5^ Germline testing also presents opportunities for cascade testing and disease prevention within families. For example, National Comprehensive Cancer Network (NCCN) guidelines recommend consideration of risk‐reducing surgery for patients with pathogenic *BRCA1*, *BRCA2*, *BRIP1*, *RAD51C*, *RAD51D*, *MLH1*, and *MSH2* germline variants based on the associated high relative risks of ovarian cancer.^6^, ^7^, ^8^, ^9^

In contrast, there are currently no targeted therapies or germline testing recommendations for AGCT. A recent study demonstrated that female subjects with the *CHEK2* I157T founder allele had increased risk of AGCT.^10^

We recently identified two patients with confirmed pathogenic germline variants in *ATM* and *CHEK2* who were undergoing treatment for recurrent AGCT, leading to an interest in examining sequencing data from a larger cohort. A clearer understanding of the role of germline mutations in AGCT oncogenesis and disease biology could create an opportunity for familial cascade testing and risk reduction strategies. We hypothesized that germline‐oriented analysis of tumor‐only Foundation Medicine testing of a large ACGT cohort could identify potential germline pathogenic variants that may contribute to AGCT risk.^11^

## 2 METHODS

### 2.1 Design, setting, and participants

This is a retrospective cross‐sectional study (Figure S1) including all granulosa cell tumor (GCT) specimens submitted for comprehensive genomic profiling (CGP) via FoundationOne®CDx (*n* = 290), FoundationOne (*n* = 224), and FoundationOne®Liquid CDx (*n* = 2) between 2012 and 2022 at Foundation Medicine, Inc. in Cambridge, Massachusetts and Morrisville, North Carolina. FoundationOne, FoundationOne®CDx, and FoundationOne®Liquid CDx are CGP assays for solid tumors, with FoundationOne and FoundationOne®CDx using a tissue sample, and FoundationOne®Liquid CDx using a liquid biopsy. All patients and specimens analyzed are from US‐based institutions. Analysis was focused on tumors with a histologic diagnosis of granulosa cell tumor that also harbored a *FOXL2* p.C134W mutation characteristic of AGCT. Samples were deduplicated using SNP matching. The study was approved by the Western Institutional Review Board (IRB) and the Medical College of Wisconsin IRB and was reported in accordance with the STROBE (Strengthening the Reporting of Observational Studies in Epidemiology) guidelines.^12^

### 2.2 DNA sequencing and processing

CGP was performed on formalin‐fixed, paraffin‐embedded tissue in a Clinical Laboratory Improvement Amendments (CLIA)‐certified, College of American Pathologists (CAP)‐accredited, New York State‐approved laboratory, as previously described.^13^, ^14^ Age, sex, and oncologic diagnosis were abstracted from requisition forms submitted by ordering clinicians. For tissue samples, test requisition forms, pathology reports, and H&E‐stained slides were reviewed by a board‐certified pathologist to confirm the provided diagnosis. Tumor sections were macrodissected to achieve a minimum of 20% estimated tumor nuclei.

DNA was extracted, quantified, and enriched via adaptor ligation hybrid capture for all coding exons of up to 324 cancer‐related genes, depending on the specific bait set, and select introns from genes frequently rearranged in cancer. Sequencing of captured libraries was performed on the Illumina HiSeq platform to a mean exon coverage depth of targeted regions of >500× for tissue samples and >2000× for liquid biopsies. Processed and aligned reads were analyzed for single nucleotide variants, indels, rearrangements, and copy‐number alterations, as previously described.^13^

### 2.3 Classification of potential germline pathogenic variants

A pre‐defined group of cancer susceptibility genes was evaluated (*ATM*, *BRCA1*, *BRCA2*, *BRIP1*, *CHEK2*, *MLH1*, *MSH2*, *MSH6*, *PALB2*, *PMS2*, *RAD51C*, and *RAD51D*). Assayed genes varied slightly based on the test performed, as the FoundationOne®Liquid CDx and FoundationOne® CDx assays report on more genes than the FoundationOne assay. For example, *RAD51C* and *RAD51D* were interrogated in 292 samples due to only being reported on the FoundationOne®Liquid CDx and FoundationOne® CDx assays. The additional 10 genes were interrogated in all 516 cases given their reporting on all three assays. To identify potential germline pathogenic variants, base substitutions and short indels reported by Foundation Medicine testing in these 12 cancer susceptibility genes were first filtered using a sensitivity‐optimized variant allele frequency (VAF) threshold of >30%. While germline variants are most likely to have a VAF of 50%, 30%–70% is a commonly accepted threshold.^15^ Next, variants were filtered based on their classification in ClinVar.^16^ Variants classified in ClinVar as “Pathogenic,” “Pathogenic/Likely Pathogenic,” or “Likely Pathogenic” by more than one submitter or by an expert panel were retained. Variants meeting VAF and ClinVar filtering criteria were considered as potential germline pathogenic variants: pathogenic variants of suspected germline origin.

VAF analysis can be used to predict biallelic loss of function of tumor suppressor genes. Mechanisms of biallelic loss of function detectable via VAF analysis include deletion of the second copy and copy neutral loss of heterozygosity (LOH). To determine whether AGCT subjects with potential germline pathogenic DNA repair gene variants fit this model, we compared the VAF of the DNA repair gene mutation to the VAF of the clonal *FOXL2* p.C134W mutation.

Predominant genomic patient ancestry was inferred from tissue specimens using a SNP‐based classifier that has been previously described.^17^

### 2.4 Clinical and family history analysis of selected patients

Clinical, pathologic, and family histories of two AGCT patients at the Medical College of Wisconsin known to have confirmed germline variants were abstracted via review of medical and genetic counselor documentation.

### 2.5 Statistical analyses

The odds ratio and two‐sided Fisher exact test *p*‐value for GCT risk for patients with the identified potential germline mutations was calculated using gnomAD v2.1 non‐cancer subjects as the control group.^18^, ^19^ Carrier counts were tallied, and the median allele number was used to determine the size of the control population.

## 3 RESULTS

### 3.1 Genomic landscape of alterations in 595 AGCT samples

**FIGURE 1:**
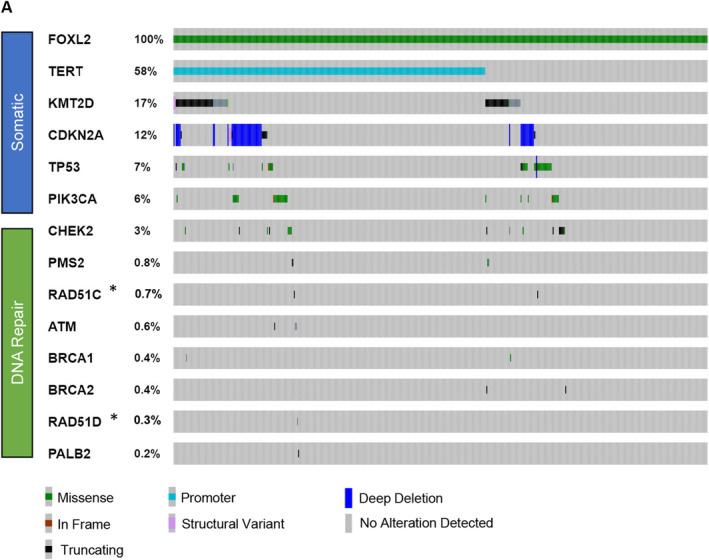
Tile plot depicting selected somatic (upper group) and predicted germline, pathogenic alterations (lower groups) in 516 *FOXL2* p.C134W‐positive AGCT samples. Each column represents a single patient. 6.6% of samples had mutations in DNA repair genes that were predicted to be germline and pathogenic, most commonly in *CHEK2* (3.48%, 18/516). *These genes were not tested on all bait sets included in this study. Reported percentages reflect the number of patients tested for the indicated gene.

A total of 595 cases with a diagnosis of AGCT were identified (Figure S1). A *FOXL2* p.C134W mutation was identified in 86.7% of samples (516/595; Figure 1; Figure S1), and these samples were included for further analysis (Table 1). Age ranged from 25 to 87 years old with a median of 57 years. Imputed genomic ancestry analysis showed 66.9% European (345/516), 15.9% African (82/516), 12.2% admixed American (a proxy for Hispanic) (63/516), 4.7% East Asian (24/516), and 0.4% South Asian ancestry (2/516).

**TABLE 1:** Clinical summary of AGCT patients.

	Cases (%)
FOXL2 p.C134W	516/516 (100)
Age	478/516 (93)
20–29	3 (0.6)
30–39	38 (7.9)
40–49	100 (21)
50–59	132 (28)
60–69	139 (29)
70–79	54 (11)
80–89	12 (2.5)
	Median = 57
Ancestry	516/516 (100)
European	345 (67)
African	82 (16)
American	63 (12)
East Asian	24 (4.7)
South Asian	2 (0.4)

The frequency of pathogenic somatic alterations, including in *TERT* (58.3%, 301/516), *KMT2D* (16.8%, 87/516), *CDKN2A* (12.4%, 64/516), *TP53* (7.0%, 36/516), and *PIK3CA* (6.0%, 31/516) were compatible with prior observations.^3^

### 3.2 Potential germline pathogenic variants were recurrently observed in DNA repair genes, especially CHEK2

A potential germline pathogenic variant was identified in 6.6% of AGCTs. *ATM*, *BRCA1*, *BRCA2*, *CHEK2*, *PALB2*, or *PMS2* variant were seen in 30/516 patients, and 3/292 patients had a *RAD51C* or *RAD51D* variant (Figure 1; Table 2). The median age of patients with a potential germline pathogenic DNA repair variant was 56, similar to the median of 57 for the cohort overall. The majority of patients were of European ancestry.

**TABLE 2:** Potential germline variants in DNA repair genes, age, and ancestry of AGCT patients.

Gene	Potential germline variant	VAF (%)	FOXL2 VAF (%)	Age at time of testing	Ancestry
ATM	R250*	47.8	44.2	74	EUR
	R1875*	53.0	45.0	35	EUR
	Splice site 8988‐1G>A	35.7	41.3	57	EUR
BRCA1	R1203*	48.9	37.9	67	EUR
	R1699Q	41.1	21.6	55	AFR
BRCA2	F1546fs*22	49.5	32.2	N.A.	EUR^a^
	S1982fs*22	47.8	50.0	56	EUR
CHEK2	I157T	50.6	48.2	43	EUR
	I157T	51.4	39.1	50	EUR
	I157T	52.0	40.0	50	EUR
	I157T	95.3	48.8	61	EUR
	I157T	50.9	35.8	61	EUR
	I157T	90.1	47.5	72	EUR
	I157T	95.6	50.8	72	EUR
	T367fs*15 (c.1100delC)	39.0	51.0	33	EUR
	T367fs*15 (c.1100delC)	47.0	59.0	43	EUR
	T367fs*15 (c.1100delC)	77.0	44.6	62	EUR
	T367fs*15 (c.1100delC)	47.8	42.0	62	EUR
	T367fs*15 (c.1100delC)	38.3	43.2	66	EUR
	S428F	88.0	46.0	39	EUR
	Splice site 444+1G>A	70.6	32.2	N.A.	EUR^a^
	Splice site 444+1G>A	69.3	42.0	54	AMR
	E479fs*3	48.3	46.4	25	EUR
	R519*	50.5	40.8	55	EUR
	Splice site 793‐1G>A	49.4	87.6	60	EUR
PALB2	Splice site 3202‐1G>A	31.8	55.1	76	AFR
PMS2	S46I	47.6	38.7	53	EUR
	D414fs*44	40.0	47.0	66	AFR
	C551*	48.0	80.0	50	EUR
	W841*	44.8	40.8	55	AFR
RAD51C	Splice site 145+1G>A	48.6	96.4	73	EUR
	Splice site 965+1G>A	41.0	24.5	62	EUR
RAD51D	Q219*	43.0	40.0	56	EUR
				Median: 56	

^a^ Indicates same patient.

**FIGURE 2:**
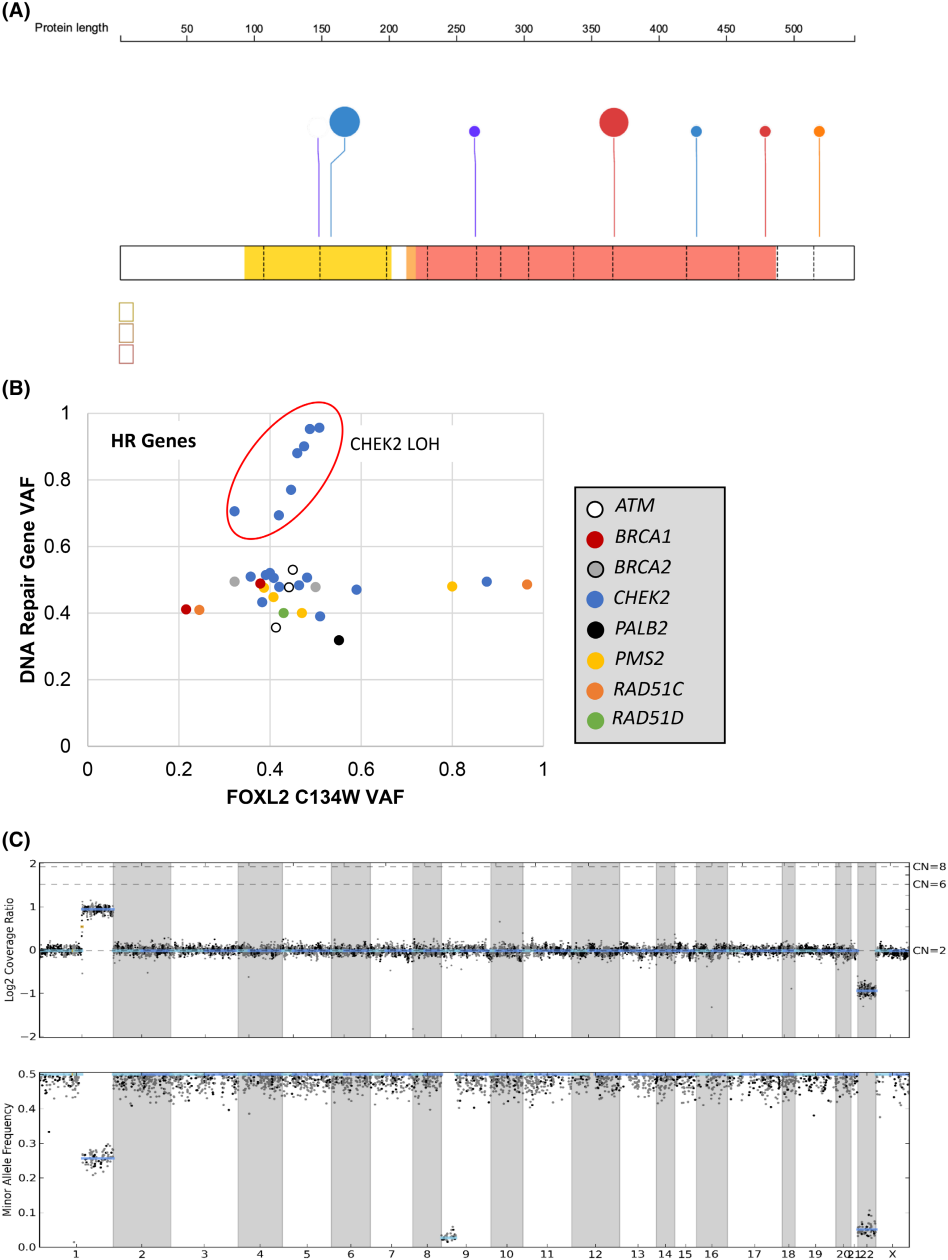
Analysis of predicted germline variants DNA repair genes, including *CHEK2*, GCT samples. (A) Predicted germline, pathogenic *CHEK2* alleles were most frequently the common founder mutations I157T and T367fs. (B) Scatter plot comparing *FOXL2* C134W VAF versus VAF for predicted germline, pathogenic mutations in selected DNA repair genes. *FOXL2* C134W is the key oncogenic driver of most GCTs, and as such, it is presumably clonal in nearly all cases. Interestingly, 39% (7/18) of cases with a *CHEK2* alteration exhibited an elevated CHEK2:FOXL2 VAF ratio (red circle), indicating loss of heterozygosity at the *CHEK2* locus. (C) Representative copy number plot demonstrating single copy loss and accompanying loss of heterozygosity at the *CHEK2* locus on chr 22, resulting in high VAF of the residual pathogenic variant of predicted germline origin.

Potential germline pathogenic variants were most frequently observed in *CHEK2*, overall identified in 3.5% (18/516) of *FOXL2*‐mutated AGCTs. There were two predominant potential germline pathogenic mutations (Figure 2A), p.I157T (38.9%, 7/18) and p.T367fs*15 (c.1100delC; 27.8%, 5/18). These were common due to founder effects in European populations. All subjects harboring these alleles had genome‐wide SNP profiles that clustered with subjects of European ancestry from the 1000 Genomes Project. Other potential germline pathogenic variants in *CHEK2* were less common (Table 2). Using gnomAD non‐cancer subjects as a control group, potential germline pathogenic *CHEK2* variants were associated with a 2.8‐fold increased risk (95% CI 1.8–4.6, *p* < 0.01) of AGCT.

Potential germline pathogenic variants were rarer in other DNA repair genes (Figure 1; Table 2). *PMS2* and *ATM* alterations were identified in 0.8% (4/516) and 0.6% (3/516) of cases, respectively. Potential germline pathogenic variants in *BRCA1*, *BRCA2*, *PALB2*, *RAD51C*, and *RAD51D* were each present in one or two individual subjects.

Excluded patients (those without a *FOXL2* mutation and therefore possibly representing juvenile granulosa cell tumors) were examined as well (Table S2). Of those, 26.9% were found to have a potential germline pathogenic variant in a rare syndrome‐associated gene (i.e., *DICER1*, *BAP1*, *IDH1*, and *STK11*). *CHEK2* potential germline pathogenic variants were also seen in 2.5% of this population.

### 3.3 Loss of heterozygosity was seen in CHEK2 potential pathogenic germline variants in AGCT

Comparisons of the VAF of the DNA repair gene mutation to the VAF of the clonal *FOXL2* p.C134W mutation are shown in Figure 2B. Most potential germline pathogenic DNA repair gene variants had VAFs that were slightly higher than the somatic *FOXL2* mutation, consistent with monoallelic status for both alterations in the setting of very high median tumor purity of 70% in this cohort.^20^ In three samples, the *FOXL2* VAF was much higher than the DNA repair gene VAF, consistent with loss of heterozygosity (LOH) at the *FOXL2* locus, but not at the DNA repair gene locus. Strikingly, 38.9% (7/18) of samples with *CHEK2* potential germline pathogenic variants had much higher VAF for the *CHEK2* allele compared to the *FOXL2* mutation in the same sample, suggesting that *CHEK2* is under LOH in those samples. Indeed, examination of these samples showed copy loss of chromosome 22 (example in Figure 2C). Of note, LOH of the remaining *CHEK2* allele is also not always observed in breast cancers with confirmed germline *CHEK2* variants.^21^, ^22^ No evidence for LOH was observed for potential germline pathogenic variants in any of the other DNA repair genes investigated.

### 3.4 Case studies of patients with potential germline pathogenic variants from tumor CGP confirmed on dedicated germline testing

**FIGURE 3:**
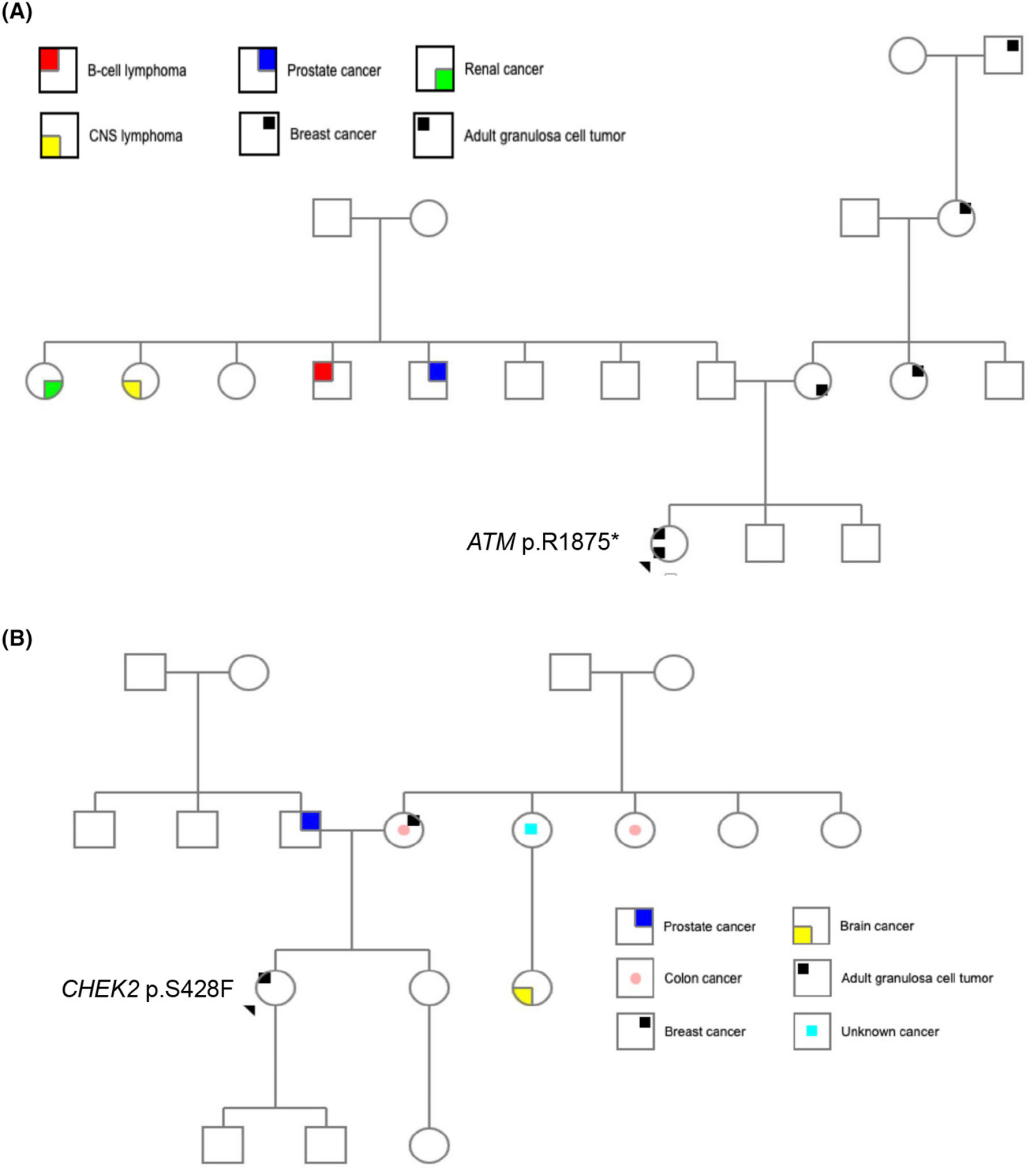
Pedigree for two patients known to harbor germline mutations and who have documented pedigree information accessible to the investigators. (A) Pedigree for patient with a germline *ATM p*.R1875* mutation. (B) Pedigree for patient with a germline *CHEK2* mutation at p.S428F.

Two patients from this study had clinical history and pedigree information available. The first of these patients was originally diagnosed with stage IC AGCT in 2013 at age 32, and subsequently received multiple surgeries and 5 different lines of treatment. Tumor CGP was performed to investigate additional treatment options, revealing an *ATM* p.R1875* mutation present at a VAF of 48.2%. Though the patient did not meet contemporaneous NCCN criteria for genetic testing based on her family history, she was referred for follow‐up genetic counseling and testing due to her *ATM* mutation detected on tumor CGP. A detailed family history revealed multiple relatives with cancer, including multiple with breast and prostate cancer (Figure 3A). Dedicated hereditary cancer gene panel testing confirmed that the *ATM* p.R1875* pathogenic variant was germline in origin. She was treated subsequently with the PARP inhibitor olaparib, on which disease progressed after 13 months.

The second patient was diagnosed with stage IC AGCT in 2007 at age 50. In 2013 she had disease recurrence and underwent secondary cytoreduction; she received multiple surgeries, tumor ablations and 4 lines of systemic therapy. Her 2013 tumor specimen was eventually sent for tumor sequencing in 2018. In 2018, after a *CHEK2* p.S428F mutation was identified at a VAF of 13.3%, lower than guidelines often recommend for secondary findings of potential germline pathogenic variants. Genetic counseling was, however, recommended by NCCN guidelines based on her family history, regardless of tumor profiling results (Figure 3B).^23^ Dedicated germline testing confirmed a germline origin of the *CHEK2* p.S428F mutation. Liver metastases were ablated again in 2019 and she underwent a third cytoreductive surgery in 2020. In 2021, she started triplet antihormonal therapy with an anti‐androgen, aromatase inhibitor and GnRH agonist.

## 4 DISCUSSION

There is no current recommendation for germline genetic testing in AGCT patients. In this study, we identified a 6.6% (30/516) frequency of potential germline pathogenic variants in this population. Within this group, we identified an elevated incidence of *CHEK2* mutations in AGCT patients. A cluster of *ATM* mutations was also observed in 4/516 patients, but comparison with the frequency in gnomAD non‐cancer subjects did not reach statistical significance. Odds ratios were not calculated for all genes due to the infrequent observations of potential pathogenic germline findings in most genes and consequently wide confidence intervals.

CHK2 kinase is a protein in the double‐stranded DNA damage response pathway. Carriers of pathogenic germline *CHEK2* variants have an increased risk of breast, prostate, colon, kidney, thyroid, and potentially other cancers.^6^, ^24^, ^25^ Additionally, augmented surveillance for breast and colon cancer is recommended.^24^ While numerous germline variants within the *CHEK2* gene have been described, the two most widely studied are the founder mutations p.T367fs*15 (c.1100delC) and p.I157T.^25^ Current cancer risk estimates and management guidelines are based on the *CHEK2* 1100delC frameshift mutation. The risks associated with missense mutations such as the I157T are lower and the clinical significance remains uncertain.^26^ A definitive ovarian cancer risk has not yet been established.

Recently, Svajdler, et al. examined 76 female patients with known *CHEK2* germline variants, and in those with ovarian cancer, 36% had an AGCT (as compared with 1.3% of ovarian cancer patients in the general population). They then examined the prevalence of the two *CHEK2* founder mutations (p.I157T and c.1100delC) in AGCT patients and found a positive association with a prevalence ratio of 26.52 (CI95:12.55–56.03) compared to the global population.^10^ Our findings support this result. We also saw increased *CHEK2* VAF in 7 of 18 *CHEK2* potential germline pathogenic variants, suggesting some loss of heterozygosity. However, chromosome 22 loss is common in AGCT and seen in 40% of 22 sequenced tumors one study^27^, ^28^, and it is unclear whether this recurrent deletion is related to *CHEK2*.

While targeted treatments for ovarian cancer patients with *CHEK2* mutations have not been prospectively confirmed, the presence of a DNA repair deficiency is in accordance with known hallmarks of cancer, and highlights possible utility of platinum or poly (ADP‐ribose) polymerase inhibitor (PARPi). PARPi has been explored in multiple cancer types for patients harboring *CHEK* and *ATM* mutations and the results have varied.^29^ For example, while olaparib has been FDA‐approved for metastatic *CHEK2*‐ and *ATM*‐mutated prostate cancer; therapeutic benefit in these patients was suboptimal in comparison to patients harboring *BRCA1*/2 mutations.^30^ For patients with metastatic breast cancer, therapeutic benefit for patients with *CHEK2* and *ATM* mutations when treated with olaparib has not yet been demonstrated, in comparison to those with *BRCA1*/*2* and *PALB2* alterations.^31^, ^32^ Further studies are needed to elucidate the potential efficacy of PARPi or other targeted therapies as treatment for AGCT with DNA repair deficiency.

Current guidelines for patients with epithelial ovarian cancer include recommendations for both somatic and germline testing.^4^, ^6^, ^33^ However, there are no current guidelines for testing in non‐epithelial ovarian cancers. In tumor types in which germline testing is not standard of care, tumor CGP can help identify potential pathogenic germline variants that warrant confirmatory testing. One large study found that while 15.7% of patients who underwent tumor profiling had a potential germline pathogenic variant identified on tumor‐only testing, only 40% of these findings were characteristic of the patient's known cancer diagnosis, making the majority of detected inherited risk alleles incidental findings.^34^ Patients in our study without LOH in their potential germline variant may represent this population. A prior analysis of 104 potential pathogenic germline *CHEK2* c.1100delC variants detected on FoundationOne testing found that the variant was reported outside of breast and colorectal cancers 66% of the time (69/104).^35^ These off‐tumor incidental findings are not only common in moderately penetrant genes such as *CHEK2*, but have been described for highly‐penetrant genes such as *BRCA1*/*2*, as well.^36^ The ability of tumor CGP to incidentally identify both on‐tumor and off‐tumor potential germline pathogenic variants can be seen as complimentary to existing genetic counseling and testing referral algorithms. This has the potential to impact not only patients' therapeutic options and personal cancer screening, but also options for cascade testing and risk stratification of family members.

Strengths of this study included a large sample size for patients with a rare tumor, and comprehensive genomic profiling of the tumor. We were also able to restrict our study sample to a high‐confidence set of AGCT patients with a confirmed *FOXL2* C134W mutation. Limitations of this study included the likelihood of a biased sample, as patients with advanced or refractory disease may be more likely to undergo tumor CGP. In addition, the potential germline pathogenic variants identified in this exploratory study are suspected germline variants, and dedicated germline testing would be required to confirm germline origin of these pathogenic variant. Outside of the two patient case studies highlighted, it is unknown whether confirmatory germline genetic testing and/or referral to genetic counseling was pursued. Two algorithmic limitations to the filtration of potential pathogenic germline variants are important to consider. First, ClinVar as likely pathogenic or pathogenic mutations in cancer susceptibility genes with VAFs less than 30% are excluded from analysis. Second, rearrangements and copy number alterations are found in a minority of hereditary cancer syndrome patients https://doi.org/10.1002/humu.22938 but are not systematically assessed for germline versus somatic origin on this analysis platform and are therefore excluded from this study.^37^ Given only short variant mutations are included this analysis, other types of potential germline pathogenic variants in the assessed genes are underrepresented. Odds ratio estimates using gnomAD as a control population are confounded by differences in populations and the fact that some subjects who were accrued for non‐cancer studies in the gnomAD cohort may in fact have cancer. Other limitations of gnomAD comparisons are that the genomic ancestry is imputed genomic ancestry, but cannot be cross‐referenced with patient self‐reported information. Imputed ancestry has been shown to be more accurate in Black and White patients than in Mediterranean or Hispanic ethnicity patients.^38^

Little has been known about germline variants contributing to AGCT. While the identified incidence of germline variants was lower in *FOXL2* C134W‐mutated AGCT patients than the epithelial ovarian cancer population, we believe that this association merits further prospective evaluation. If the association is confirmed, women with AGCT could be included in genetic testing referral algorithms. Because organizations such as the NCCN, Society of Gynecologic Oncologists (SGO) and American Society of Clinical Oncology (ASCO) greatly influence genetic testing referral algorithms, this and other similar studies should be taken into consideration when updating practice guidelines pertaining to germline genetic testing.

## AUTHOR CONTRIBUTIONS

**Rebekah M. Summey:** Methodology (supporting); visualization (supporting); writing – original draft (lead); writing – review and editing (equal). **Brennan Decker:** Conceptualization (equal); data curation (equal); formal analysis (equal); investigation (equal); methodology (equal); supervision (equal); visualization (equal); writing – original draft (equal); writing – review and editing (equal). **Erica Gornstein:** Conceptualization (equal); data curation (equal); formal analysis (equal); methodology (equal); writing – original draft (equal); writing – review and editing (equal). **Kali C. Dougherty:** Writing – original draft (equal); writing – review and editing (equal). **Janet S. Rader:** Conceptualization (equal); investigation (equal); methodology (equal); supervision (equal); writing – review and editing (equal). **Elizabeth Hopp:** Conceptualization (equal); investigation (equal); methodology (equal); supervision (equal); writing – original draft (equal); writing – review and editing (equal).

## FUNDING INFORMATION

No outside funding was received for the completion of this project.

## CONFLICT OF INTEREST STATEMENT

RS, JR, and EH have no disclosures. EG is employed by Foundation Medicine and holds stock in Roche. BJD is employed by Foundation Medicine and holds stocks and stock options in Roche. KD is employed by Foundation Medicine, received honoraria from the National Society of Genetic Counselors, and holds stock in Roche and Myriad Genetics.

## ETHICS STATEMENT

The study was approved by the Western Institutional Review Board (IRB) and the Medical College of Wisconsin IRB, and was conducted in concordance with the Federal Policy for the Protection of Human Subjects.

## CONSENT

This project was retrospective and information is de‐identified, thus it was granted a waiver of informed consent.

## PERMISSION TO REPRODUCE MATERIALS FROM OTHER SOURCES

N/A.

## CLINICAL TRIAL REGISTRATION

N/A.

## Supporting information


Data S1.

## Data Availability

Data is not available for public use since it is proprietarily held by Foundation Medicine.
