# Supplementary material for: Landscape of potential germline pathogenic variants in select cancer susceptibility genes in patients with adult‐type ovarian granulosa cell tumors
Source: Cancer Med. 2024 Jun 19;13(12):e7340. doi: 10.1002/cam4.7340 (PMC11187164; doi:10.1002/cam4.7340)

**Table S1.** AGCT risk assessments for *CHEK2* using different publicly available population data as controls (gnomAD non-cancer subjects vs the CARRIERS study) and different definitions of *CHEK2* carrier status (all variants meeting criteria for pathogenic, potentially germline versus only for founder alleles).

|  |  |  |  | **aGCT *CHEK2* Carrier Definition** | | | | | | | |
| --- | --- | --- | --- | --- | --- | --- | --- | --- | --- | --- | --- |
|  |  |  |  | **Pathogenic, Potentially Germline** | | |  | **p.I157T and c.1100delC Founder Alleles** | | | |
|  |  | **Total n** |  | **n (prevalence)** | **OR  (95% CI)** | **P-value** |  | **n (prevalence)** | | **OR (95%CI)** | **P-value** |
|  | aGCT | 516 |  | 18 (3.5%) | NA | NA |  | 12 (2.3%) | NA | | NA |
|  |  |  |  | |  |  |  |  |  | |  |
| **Control Population** | gnomAD Non-Cancer | 118,515 |  | 1,491 (1.3%) | 2.8  (1.8-4.6) | P<0.001 |  | 1,491 (1.3%) | 1.9  (1.1-3.3) | | P=0.032 |
|  | CARRIERS | 32,544 |  | 138 (0.4%) | 8.5  (5.2-14.0) | P<0.001 |  | 138  (0.4%) | 5.6  (3.1-10.2) | | P<0.001 |

**Table S2**: Potential germline variants in rare syndrome-associated genes, age, and ancestry of GCT patients. 1. Indicates same patient; # Indicates patient without FOXL2 C134W mutation, all other patients have FOXL2 C134W.

| **Gene** | **Potential Germline Variant** | **Age at Time of Testing** | **Ancestry** |
| --- | --- | --- | --- |
| BAP1 | R300fs*6 | 57 | EUR^1#^ |
|  | S628fs*8 | 57 | EUR^1#^ |
| DICER1 | Q249* | 58 | AMR^2#^ |
|  | E1705K | 58 | AMR^2#^ |
|  | E1705K | 17 | EUR^#^ |
|  | D1810V | 44 | AMR^#^ |
|  | C1333* | 33 | AFR^3#^ |
|  | E1813G | 33 | AFR^3#^ |
|  | D1437fs*16 | 78 | EUR^4#^ |
|  | E1813D | 78 | EUR^4#^ |
|  | E1813D | 13 | AMR^#^ |
|  | E1813K | 25 | EUR^#^ |
|  | E1813Q | 23 | EUR^#^ |
|  | R187* | N.A. | EUR^5#^ |
|  | E1813Q | N.A. | EUR^5#^ |
| FLCN | H429fs*39 | 82 | EUR |
|  | Y463* | 48 | EUR |
| IDH1 | R132C | 17 | AFR^#^ |
| STK11 | L67P | 64 | AFR^#^ |
|  | Q220* | 68 | EUR |
|  |  | Median: 44 |  |


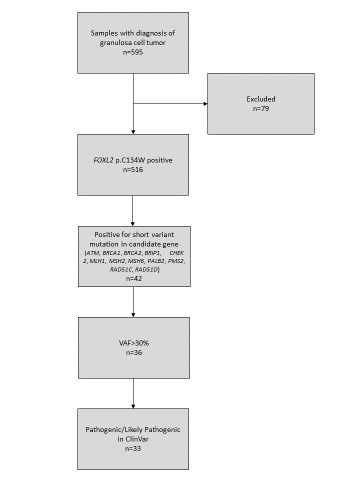

Supplement: Supplementary file 1 — Data S1. [file CAM4-13-e7340-s001.docx]
